# Professional Underbidding in England

**Published:** 1834

**Authors:** 


10. *Professional underbidding in the West of England.*— Should any one doubt our English origin, we might refer to to an article in the London Medical Gazette, on the state of the profession in the West of England, to prove that many of the physicians in the Valley of the Ohio, have descended from that celebrated nation. The picture which it presents of underbidding labors for the acquisition of business, would apply with some little variation, to certain members of the profession even here, within the dominion of the boasted Queen of the West! We shall give an extract which may serve to keep some of our brethren in countenance. It is from the pen of Mr. Tomkins, surgeon in Yeovil.

"Parishes are usually let by tender, and the lowest bidder generally gets the appointment to take charge of them. The low rate at which persons are found to accept them is truly disgraceful to the members of a respectable profession. I will name a few particular itstances to prove the fact; assuring you, at the same time, I could cite a hundred similar ones. The parish of East Coker, Somerset, containing upwards of 1400 persons, the majority of whom are paupers, is attended by a practitioner—a Mr. Hansard, of Montacute—residing four miles from the place, at a salary of 12*l.* per year; and for this sum he includes all medical and surgical cases, accidents of every description, small pox, inoculation, or vaccination, and difficult midwifery cases, together with attendance on coroners' inquests, lunatics, &c. Mr. Hansard stipulates to attend regularly at the poor-house three times a week, independently of all other calls; and, on a moderate calculation, his turnpikes must cost him at least three pounds in the twelve months; such being the usual terms of agreement where parishes are compounded for. The parish of East Chinnock is attended by a practitioner—a Mr. Gerrard, of Crewkerne—residing five miles distant; who compounds for all cases at 8*l.* per year. The population is about 1000, and by far the greater number are paupers. Mr. Gerrard has to pay two turnpikes every journey he takes. The parish of Piddle-Trenthide, Dorset, is attended by the Messrs. Davis, of Cerne, residing at a distance of four miles, at a salary of 10*l.* per year. The population is upwards of 800. The sum paid for medical and surgical attendance on the poor of the same parish, in the year 1814, was 70*l.*; but owing to the disgraceful system of letting by tender, it has been reduced to the sum above stated. The parish of Stoke Sub-Hampdon, Somerset, is attended by a Mr. Westcott, of Martock, residing two miles distant, and he compounds for every case that may occur, agreeing to find medicines and surgical instruments, at a salary of 8*l.* per year: the population is upwards of 1600. There are a vast number of paupers at Stoke, and they are very liable to serious accidents, in consequence of their being employed to quarry and hew stones, which are used in almost every building of consequence within twenty miles of the place. The parish of Norton Sub-Hampdon consists of a population of 800 persons; the paupers are numerous, and generally employed at the stone-quaries; the poor are attended by a Mr. Stuckey, of Martock, who compounds for all cases at a salary of 5*l.* per year: the distance of Norton from Martock is three miles. Chisselborough, a parish four miles distant from Martock, is also attended by Mr. Stuckey, and every thing compounded for at 5*l.* per year: there are seven or eight hundred people, and the paupers are principally employed at the Hampdon stone-quarries."

All this would seem incredible, but it is avouched by five gentlemen of Mr. Tomkins' neighborhood, the whole of whom, like himself, are members of the Royal College of Surgeons, and appear before us, therefore, as respectable and credible witnesses.

The following anecdote, related by Mr. T., is calculated to remind many of us, of cases in which we have been compelled, not merely to give our professional services gratis, but actually to advance money for the support and comfort of our patients.

"I cannot here omit to mention one fact, which, some time since, happened to a respectable surgeon in this neighborhood. He was sent for to a poor man who had met with a very serious accident, and on his arrival found him by the road side. He directly said, "I can do nothing for the man here, you must take him to the inn;" which was near the spot, and in the parish where the injury was received. The accident was such that the surgeon was obliged to attend to it without delay, and the man remained at the inn many weeks afterwards, before he could be removed to a distant parish, where he belonged. After his recovery, the surgeon sent his bill to the overseers of the parish where the accident happened, and they resisted payment, as they had not given an order. The surgeon then applied to the parish where the man belonged, and they also resisted payment; pleading that they were not liable, as they had given no order to the medical attendant, and the accident happened in another parish. The surgeon found, that as he had acted without an overseer's order, he could not recover his charges by law, and the individual himself was unable to pay him. This, however, was not all; the innkeeper was refused payment by both parishes for the keep, attendance on, and nursing this pauper; and not being in a situation to lose his expenses, he naturally inquired who sent the man to his house; and finding that the surgeon had ordered him there, and given directions for his food, nurses, &c., he actually brought the charge on him, and the surgeon was compelled to pay the whole expense."

The medical is, perhaps, the only profession, except that of the itinerant show-man, which may not be expurgated of quackery, to a tolerably decent degree. Credulity, parsimony, love of mystery, admiration of impudence, a desire to be imposed upon, and a suspicion of every thing which belongs to regular science, are principles too deeply rooted in the human mind to be easily eradicated; and while *they* are in operation, we shall see empyricism prosperous, and the dealers in low charge, preferred to those, who place their skill and services at such prices, as will give them honorable recompense, for the days and nights of protracted and anxious study, which are necessary to successful practice.

